# Should We Continue EU Cohesion Policy? The Dilemma of Uneven Development of Polish Regions

**DOI:** 10.1007/s11205-022-03048-8

**Published:** 2022-12-16

**Authors:** Maciej Jagódka, Małgorzata Snarska

**Affiliations:** College of Economics, Finance and Law, Cracow University of Economics, 31-510 Krakow, Poland

**Keywords:** Cohesion policy, Sustainable development, Human capital, Innovativeness, Wilcoxon test, E24, H10, J24, J40, O15, O30, R15

## Abstract

The European Union and the whole world are facing the problem of increasing income and wealth inequalities at the country and regional levels. Inequalities are a severe obstacle to sustainable and balanced growth. Politicians perceive this problem by trying to implement different types of development models. The cohesion policy is the European Union's response to the development differences between countries and regions. It is clearly defined that development has to be sustainable and inclusive, which is reflected in the objectives of all leading EU development strategies. The aim of this paper is (1) to examine how effective the EU cohesion policy is in reducing regional disparities (2) to assess that the main cause of regional disparities is the misallocation of human capital. The authors base their study on the regions of Poland, estimating the state of their human capital and innovation between 2004 and 2018. We examine the effectiveness of cohesion policy using functional data analysis—the Wilcoxon test, which was used for the first time for this type of issue. The current question is whether to help metropolises and large cities, which will entail the development of smaller units, or whether to try to increase the rate of development of smaller and economically weaker territorial units in the first place.

**Supplementary Information:**

The online version contains supplementary material available at 10.1007/s11205-022-03048-8.

## Introduction

This paper assesses the impact of EU accession on human capital and the innovativeness of spatial disparities within Polish regions. Cohesion policy is the EU's investment program to help regional development. It aims to reduce economic, social, and territorial disparities between EU regions, channeling investment through three main funds, namely the European Regional Development Fund, the European Social Fund, and the Cohesion Fund. Cohesion policy (CP) is widely discussed in the literature (Bachtler et al., 2017; Bachtrögler et al., 2020; Berkowitz, Monfort, & Pieńkowski, 2019; Crescenzi & Giua, 2020; Marzinotto, 2012; Mohl & Hagen, 2010; Vedrine & Le Gallo, 2021). General researchers check the link between CP effectiveness in increasing EU regional growth and reducing disparities (Boldrin & Canova, 2001; Dall’Erba & Le Gallo, 2008).

The effectiveness of EU funds is still a matter of controversy (Fiaschi et al., 2018; Maynou et al., 2016), the same as CP and its impact on growth. Some studies show positive impact (Beugelsdijk & Eijffinger, 2005; Dall’Erba, 2005; Rodriguez-Pose & Fratesi, 2004) and some negative or no significant impact (Boldrin & Canova, 2001; Breidenbach, Mitze, & Schmidt, 2019; Dall’Erba & Le Gallo, 2008; Esposti & Bussoletti, 2008). Differences in research occur because cohesion policy is heterogeneous. It includes numerous public intervention programs. The context of CP implementation is also different because of the different developmental conditions of regions. The methodology of the study, and the set of characteristics, are also essential (Bachtrögler et al., 2020; Crescenzi et al., 2016; Darvas et al., 2019). The effects of cohesion policy are often regionally differentiated, due to the specifics of the region and its level of development (Biedka et al., 2022; Lucatelli & Monaco, 2018).

The purpose of our study is to assess the impact of CP on reducing regional disparities in human capital and innovation. We consider that human capital is today the most critical factor of socioeconomic development and innovation and the explanation of inequalities. Such a study has never been done before. We believe it allows for a better assessment and recommendations on the public intervention policies applied in many aspects. Our research question is whether it is better to help metropolises by contributing to human capital migration in wealth building or to address programs for more economically vulnerable areas. Cohesion policy may be hampered by human capital migration. People make decisions to move based on better job opportunities (Haapanen & Tervo, 2012), higher wages (Di Cintio & Grassi, 2013; Faggian et al., 2007), culture (Kontuly & Smith, 1995), etc. Human capital migration favors metropolitan areas, often leading to a brain drain of people from economically weaker regions (Hoare & Corver, 2010). This is a challenge for cohesion policy and what course of intervention to use to make development more sustainable.

In what follows, we attempt to assess whether the cohesion policy applied in Poland over 15 years has helped to reduce regional disparities or not. The adopted human capital and innovativeness indexes cover the research period of years 2004–2018. We apply our research methods for Polish regions by taking data for 16 voivodeships (NUTS2). We also examined the impact of two European Union Operational Programs—Human Capital and Innovative Economy (both co-financed by the European Union), on the convergence processes in Polish regions in human capital and innovativeness. Likewise, we have been focusing mainly on EU programs addressed to human capital and innovativeness. The state of human capital was determined based on 120 characteristics defining human capital and 28 defining innovation, using the TOPSIS composite index weighting method (considering five independent methods of synthetic index creation). A detailed description of how the index was built is available in the: (Jagódka, 2021; Jagódka & Snarska, 2021). We examine the effectiveness of CP using functional data analysis-local and global Wilcoxon tests. The concept of data depth used here aptly assesses the similarity of the development paths of different regions with the chosen time horizon, clearly indicating whether the distances of these paths have increased or not. Overcoming regional disparities is crucial to economic stability and cohesion. Poland, as a developing country, represents the former Eastern Bloc countries. The East–West dualism appears either for human capital or innovativeness in Poland, either due to historical and territorial aspects of country (Czyż & Hauke, 2011; Gorzelak, 2006; Gurgul & Łach, 2019; Opiłowska, 2019; Wielki et al., 2018).

The remaining part of this paper is organized as follows. Section 1 gives an overview of related literature describing European Cohesion Policy, and the link between human capital, innovativeness, economic growth, and disparities. Section 2 is dedicated to materials and methods. Section 3 treats European Cohesion Policy. Section 4 presents the results and Sect. 5 discusses the empirical analysis. Section 6 is dedicated to conclusions. 

### The Link Between Human Capital, Innovativeness, Economic Growth, and Disparities

Today's global development is characterized by great diversity. High and growing spatial disparities are noticed mainly in emerging economies. In wishing to catch up, developing countries want to maintain a high-growth rate, which unfortunately often devastates the environment and increases internal inequalities in spatial, social, and economic areas. The first explanation of this state of affairs is economic geography, i.e., first and second nature geography. First nature geography says that some regions are privileged because of their proximity to rivers, natural resources, ports, and borders. Many of these factors are responsible for the success of, for example, coastal countries or those located in more favorable climate zones. On the other hand, second nature geography emphasizes several interactions that occur between actors and, in particular, the growing profits generated by being created by large agglomerations. Cities tend to create high productivity, and agglomeration forces act to create a spiral of self-perpetuating growth. However, it is crucial to determine the strength of this development, as it depends on many aspects of economic life, such as the quality of human capital, infrastructure, state efficiency, openness to trade, and the political and legal environment. By understanding the overall relationships, spatial differences can be explained (Kanbur & Venables, 2005; Kanbur et al., 2006; Lang, Henn, Ehrlich, & Sgibnev, 2015).

Human capital research has been going on for a long time, but the main precursors of the human capital theory of the 1960s are considered to be three authors: Mincer (1984), Schultz (1961), and Becker (1994). Their theoretical considerations and empirical research became the basis of human capital theory, which is still developed in the social sciences today. Human capital is a complex economic category that explains why technical progress through innovation is possible in some countries and not in others. The OECD’s definition of human capital captures the essence of the issue. Human capital consists of knowledge, competencies, skills, and other attributes that enable an individual to build personal, social, and economic well-being (Healy & Côté, 2001).

According to Statistics Poland (SP), innovation resulting from human capital is enterprises’ ability to create and implement innovations and the current ability to introduce new and modernized products and modified or new technological or organizational and technical processes. This definition was proposed by the international methodological standard called the Oslo Manual (2018).

The positive link between human capital and economic growth has been proven at the country level as well as at the regional level (Badinger & Tondl, 2003; Biscaia et al., 2017; Di Liberto, 2008; Engelbrecht, 2002; Fagerberg et al., 1997; Sterlacchini, 2008). One comprehensive study on the determinants of regional growth was Gennaioli et al. (2013). The authors collected data for 1569 regions from more than 100 countries. Human capital is expanded through regional education, which refers to the education of workers and entrepreneurs. Taking care of the regional education system increases the quality of the factors embodied in people. According to Lucas (2002), the main factor of growth is the accumulation of human capital in knowledge. Differences in the level of national income between countries should be seen precisely in the differentiation of this resource. Human capital formation occurs in schools, research, development units, production, and trade. Periods of the economy's rapid growth can be associated with accumulating knowledge and work experience during work. The relationship between knowledge accumulation and economic growth is also indicated by other researchers (Landes, 1990, 2000). Families, schools, and businesses release human capital (Heckman & Jacobs, 2013). Educated entrepreneurs increase supply by starting businesses and increasing their productivity. Economic development is especially evident in creative entrepreneurs' productive concentration places, mostly educated people and well-trained workers. Economic growth must lead to integrated development.

Since human capital is the primary determinant of innovativeness, regional differences in innovation potential are, to no small extent, a derivative of the spatial distribution of human capital (Bronzini & Piselli, 2009; Rodríguez-Pose & Vilalta-Bufí, 2005). Human capital is a significant driver of regional growth. It provides insight into the degrees of regional disparity (Erdem, 2016). Since human capital is considered a major determinant of regional inequality and a catalyst for innovation and growth, it is important to look at whether cohesion policy equalizes the distribution of human capital and innovation within regions. This is crucial for the public policies implemented in the EU. Cohesion policy is designed to help achieve sustainable development (SD). The definition of SD has evolved (Sachs, 2015). Intergenerational equity, or the need to preserve resources for future generations, is one of the main features of SD. However, the only way to achieve this goal is to conceive of development as a multidimensional concept, considering economic, social, and environmental (Alaimo & Maggino, 2020). Therefore,to elaborate on sustainable development it is necessary to act holistically within the social, economic, and environmental cohesion which can be achieved through policies fostering economic growth, greater social equality, and the reduction of negative environmental impacts, the needs of current and future generations are expected to be enhanced (Guillen-Royo, 2018).

The desired directions of human capital development are achievable through the EU cohesion policy implemented at the social, economic, and territorial levels (Prusek, 2015). It is implemented over a seven-year programming and funding horizon, with €349.4 billion allocated between 2014 and 2020. The beneficiaries of help are mainly less developed European regions. Human capital occupies an important place in the CP, as it finances activities to develop this resource in cohesive countries. This results in improving the education and qualifications of the inhabitants of these countries (Zawistowski, 2011). As part of the evaluation of the effectiveness of CP measures, cyclical reports are published, the latest in 2022, the Eighth Cohesion Report (European Commission, 2022). The European Commission indicates that in the short run there are significant differences between regions within the Member States, where the estimated impact on GDP is higher than without CP. It ranges from 1.1% to 5% in Poland (European Commission, 2022). Additionally, the report is mentioned that GDP per head will be 2.6% higher in less developed regions due to help from the cohesion policy in 2014–2020. Moreover, the gap between GDP per head in regions representing the top and bottom deciles will fall by 3.5%. During the COVID-19 pandemic crisis, the EU's cohesion policy response was swift: additional funds were mobilized, making spending on the crisis response eligible and allowing higher co-financing rates. Unfortunately, public finances improved steadily until 2019, but the COVID-19 crisis reversed the trend.

In the 2007–2013 perspective in the context of human capital, the Human Capital Operational Program (HC OP) and the Innovative Economy Operational Program (IE OP) should be mentioned. National Strategic Reference Frameworks (NSRFs) were the strategic documents specifying Poland's development concerning the direction outlined in the EU programming documents. Particular importance should be attached to the HC OP, under which ca. 11.4 billion euros were allocated (9.7 billion euros are financing from the European Social Fund, and the remaining part—is national financing). The effects of achieving the objectives of this program, according to available reports, should be considered positive. The general conclusion in one of the reports is that there is a noticeable positive impact of the interventions carried out under the Human Capital Operational Program in all analyzed areas (Sochańska-Kawecka et al., 2015).

Within the framework of the regional component of HC OP, the convergence process was strengthened in the labor market and social integration. In education and adaptability, the coefficients of the convergence rate in the two cases: i.e., with the scenario with funds and without funds, are similar. In the context of institutional capacity building, the rate of convergence is even higher in the unfunded scenario. In addition, it was pointed out that weak convergence effects occurred in the regions with the worst baseline parameters, which could be due to a higher concentration of creative and entrepreneurial people in developed urban centers and regions with higher rates of development (Sochańska-Kawecka et al., 2015).

The current regional policy in human capital development is carried out in the EU Financial Perspective 2014–2020. Poland will receive approximately EUR 82.5 billion under the CP in this period (Ministry of Funds & Regional Policy, 2019). The intervention strategy of European funds in terms of three policies: cohesion, agriculture, and fisheries, was adopted in the Partnership Agreement document (Ministry of Funds & Regional Policy, 2020). The instruments for implementing the tasks set out in this plan are national operational programs (NOP) and regional operational programs (ROP). Together, they form the strategic and programmatic basis for the 2014–2020 financing perspective. The Partnership Agreement provides a reference point for detailed solutions included in the operational programs. In the current financial perspective, the share of regional programs in the total allocation was increased from 25 to 40% compared to the previous one. Regional policy in human capital development can be implemented mainly through the Operational Program Knowledge Education Development 2014–2020 (OP WER) and 16 regional operational programs. The priority areas of OP WER include various measures for the development of human capital, e.g., in the field of economy and education, higher education, effective public policies in the labor market, and youth employment (Jaźwiński, 2017).

To achieve the ambitious objectives of the European equalization policy, there is a need to develop a solid territorial rationale for structural interventions undertaken after 2020 within the framework of CP and other integrated policies (Szlachta, 2018).

## Materials and Methods

To assess the effectiveness of CP within human capital and innovativeness, we adopt the Wilcoxon test for functional data. We considered that methods based on data depth would help us to assess whether the state of human capital and innovation of the 16 Polish voivodeships in the period under study has changed concerning the Mazowieckie voivodeship or not. Wilcoxon test for functional data was used for the first time to assess the degree of development of individual regions, as we believe that the information medium obtained is wealthy and complementary to such methods as panel data. The Wilcoxon test based on the depth data was used because we treat human capital and innovation data as functions in the data space. The function models are our indexes. We treat the multidimensional space of variables and data as Hilbert L2 space or data function space. So, that is why we used the Wilcoxon test using the depth data. Our data space is a function space, and the Wilcoxon test is used to test pairs of variables (in our case functions) and see if there was a significant change in the relationships under study (human capital or innovation) over the period under study. Additionally, a detailed list of variables of human capital and innovativeness index can be shared by authors on request.

The Wilcoxon test is used to compare two measurement variables, forming so-called dependent groups. It is an alternative to a Student's t-test of two means, assuming that variables are measured on an ordinal and quantitative scale. The test's initial criterion is the possibility of ranking, i.e., maintaining a linear ordering between measurement variables. Under the algorithm used in the test, the differences between the measurements are determined within a fixed number of observations, i.e., successive values of human capital and innovativeness indices for all regions at the same time. One then orders all observations given a specific linear operator. The ranking process involves sorting data from the lowest to the highest, regardless of their differences.

Moreover, the rank sums for negative and positive differences are calculated separately. The missing values of one index in a given year exclude that entire year from the study. The more significant value of these two rank sums represents the test statistic value. The testing burden is the potential autocorrelation and non-stationarity of the time series. The test assumes the independence of observations. Its absence can distort the results obtained by inference. The hypothesis of differences in human capital and innovation between the years under study is verified. In this paper, we used an approach that limits these difficulties, i.e., a functional time series, understood as a series of observations for which the deterministic trend is eliminated.

A unit functional observation is not a point in the data space, but a function or a curve. Assuming that ${x}_{it}$ t is the value of the human capital or innovation index for the i-th province in year t, where $i=\mathrm{1,2},\dots ,16, t=\mathrm{1,2},\dots ,15$ functional data are pairs ${(T}_{it}, {y}_{it}), {T}_{it}\in [\mathrm{2004,2018}]$ which are realizations of random functions on a Hilbert space with norm ${L}^{2}$ within $\left[\mathrm{0,1}\right]$ that is a generalization of the classical Euclidean distance measure and number space to the space of functions (Ramsay & Dalzell, 1991). In functional time series, under the assumption that ${X}_{i}\left(t\right)={f}_{t}({x}_{it})$, defined as the human capital or innovation index function at time t, the functional time series is defined as ${{X}_{i}\left(t\right)=f}_{t}\left({x}_{it}\right), t\in T,i\in \{1,\dots ,16\}$, when the continuous human capital or innovation index function additionally depends on time. The Wilcoxon test for functional time series is performed when the series exhibits stationarity on the function space. Functional stationarity is a generalization of the concept of stationarity for stochastic processes and one-dimensional time series to the function space (Bosq, 2012; Horváth & Kokoszka, 2012). The functions ${f}_{t}$, describing the diversity of development paths in human capital and innovation in particular years, converge so quickly that the line describing the development of each province is considered almost identical. Deviations from the assumed path are small and random in nature, and when they occur, they are estimated using a data depth function that measures the centrality or deviation of a given point relative to the multivariate data set or probability distribution that was generated by that point.

Functional depth provides a tool for organizing functional data to show deviations from the center of the distribution while providing compensation for the potential lack of linear ordering. The depth function $D(.,F)$ and its associated vector ${f}_{t}\in H$: $D\left({f}_{t},F\right)\to [\mathrm{0,1}]$ is considered a measure of the centrality of the vector for the distribution.$F\in P\subset H$ Higher values of depth for the determined function of human capital or innovativeness mean a smaller distance of a given voivodeship to the indicated path. The Wilcoxon test allows for examining differences in multivariate functional data (Jurečková & Kalina, 2012; Kosiorowski et al., 2019; Liu & Singh, 1993; Zuo & He, 2006).

In this paper, we proposed an approach using functional time series, that is, realizations of stochastic processes on the space of Hilbert functions. The path, or development curve for human capital, respectively ${X}_{i}=\{{X}_{i}\left(t\right), t\in \left[0,T\right]\}$ and innovativeness ${Y}_{i}=\{{Y}_{i}\left(t\right), t\in \left[0,T\right]\}$, where $t=0$ is the beginning of the study, i.e., the year 2004 and $t=T$ represents the year 2018, is viewed as a random variable from the random function space ${L}^{2}(\left[0,T\right])$, where ${\mathrm{L}}^{2}$ is a space with a Borel σ algebra. In this part of the study, the relevant human capital and innovation development scenarios in each province were assigned numerical values of human capital or innovation indices. The formula expresses the product of observations in this space.$\langle x,y\rangle ={\int }_{0}^{T}x\left(t\right)y\left(t\right)dt$ The random function space includes all realized and hypothetical human capital paths or innovations during the period under study. Realized observations $i=1, \dots , 16$ were presented using indices describing development trajectories of human capital or innovation in Polish voivodeships. A development trajectory is a continuous function recorded at discrete moments, i.e., at the end of a given year t. The transformation of discrete index observations can be performed by nonparametric smoothing (Ramsay et al., 2009). One of the procedure's assumptions is the concept of strict and weak stationarity on the function space, which can be considered similarly to classical time series (Horváth et al., 2014). In this paper, two Wilcoxon tests were conducted for both human capital and innovation levels. Hypothesis verification in the global test, where $F\left({X}_{i}\left(0\right)\right)$ and $F({Y}_{i}\left(0\right))$ present the development level of provinces in 2004, and $F\left({X}_{i}\left(T\right)\right)$ and $F({Y}_{i}\left(T\right))$ the human capital status and innovation level in 2018, respectively, can be written as follows:

1 $$\begin{gathered} H_{0} :F\left( {X_{i} \left( 0 \right)} \right) = F\left( {X_{i} \left( T \right)} \right) H_{1} :F(X_{i} \left( 0 \right) \ne F\left( {X_{i} \left( T \right)} \right) \hfill \\ H_{0} :F\left( {Y_{i} \left( 0 \right)} \right) = F\left( {Y_{i} \left( T \right)} \right) H_{1} :F(Y_{i} \left( 0 \right) \ne F\left( {Y_{i} \left( T \right)} \right) \hfill \\ \end{gathered}$$

The null hypothesis means that the observations follow the same distribution, while the alternative hypothesis means they are different. In economic terms, it is said that interregional disparities in human capital development and innovation in 2004 and 2018 are expressed by distributions of respectively $F\left({X}_{i}\left(0\right)\right)$, $F\left({X}_{i}\left(T\right)\right)$ and $F\left({Y}_{i}\left(T\right)\right)$. No basis for rejecting the null hypothesis should be read as no shift in the structure of disproportions between voivodeships before and after Poland's accession to the EU (EU cohesion programs).

The local Wilcoxon test's idea is to determine moments for which changes may occur, i.e., an increase or decrease in disparities in the level of human capital development and innovation of voivodeships. The hypotheses for this part of the test can be expressed as follows:

For human capital:

2 $$\begin{gathered} H_{0} :F\left( {X_{i} \left( 0 \right)} \right) = F\left( {X_{i} \left( 2 \right)} \right) = \ldots = F\left( {X_{i} \left( T \right)} \right) \hfill \\ H_{1} : F\left( {X_{i} \left( 0 \right)} \right) = \ldots = F\left( {X_{i} \left( t \right)} \right) > F\left( {X_{i} \left( {t + 1} \right)} \right) = \ldots = F\left( {X_{i} \left( T \right)} \right) \hfill \\ \end{gathered}$$

And for innovativeness:

3 $$\begin{gathered} H_{0} :F\left( {Y_{i} \left( 0 \right)} \right) = F\left( {Y_{i} \left( 2 \right)} \right) = \ldots = F\left( {Y_{i} \left( T \right)} \right) \hfill \\ H_{1} : F\left( {Y_{i} \left( 0 \right)} \right) = \ldots = F\left( {Y_{i} \left( t \right)} \right) > F\left( {Y_{i} \left( {t + 1} \right)} \right) = \ldots = F\left( {Y_{i} \left( T \right)} \right) \hfill \\ \end{gathered}$$

where $t\in \{\mathrm{1,2},\dots 15\}$ is a specific year or years behind the changes in the structure of regional development in human capital and innovation.

Testing for human capital s done independently of testing for innovation. Hence, the moments for which structural changes were detected in subsequent years need not be the same. The global Wilcoxon statistic was applied in a moving window (the so-called rolling scheme). Determining the ordering of provinces in the space of functions is based on data depth. The ranking of functional observations is determined based on deviations from the center of the distribution, i.e., the functional median. Data depth allows for measuring the shape of the probability distribution. The objects closest to the center of symmetry of a given distribution have the most significant depth value. Simultaneously, as one moves away from the center of the distribution, nested regions of depth appear according to the pattern of topographic isolines (López-Pintado & Romo, 2009). Differences ${d}_{i}$= ${X}_{i}\left(t\right)-MED\left({X}_{i}\left(t\right)\right)$ for the functional Wilcoxon test are estimated against the averaged path of human capital development, as for innovation ${d}_{i}$= ${Y}_{i}\left(t\right)-MED\left({Y}_{i}\left(t\right)\right)$. Moreover, path averaging is done based on the functional median. Hierarchization of the resulting set of absolute deviations is performed using the adjusted band depth. The functional depth determines the probability assigned to each part of all provinces' development paths. The depth is a multivariate generalization of the histogram. The global Wilcoxon statistic takes the form:

4 $$S = \min \left( {W^{ + } , W^{ - } } \right)$$

where ${W}^{+}=\sum_{{d}_{i}>0}{R}_{i}$, ${W}^{-}=\sum_{{d}_{i}<0}{R}_{i}$ are the statistics corresponding to the sum of the ranks for positive and negative deviations, respectively, from the depth-induced median, that is, the index values at which the depth function reaches its maximum. The ranks are obtained using the statistical depth of the data $D(.,F)$, for each province ${X}_{i}\left(t\right)\in {R}^{d}$ in a fixed year, t defines a measure of centrality $D\left({X}_{i}\left(t\right), F\right)\in [\mathrm{0,1}]$, assuming a probability distribution $F\in P$ on the feature space ${R}^{d}$ or an empirical distribution ${F}_{n}\in P$. The ranks are determined based on the statistical depth of the data $D(.,F)$, which for a fixed year t allows for each province ${X}_{i}\left(t\right)\in {R}^{d}$ to assign a centrality measure $D\left({X}_{i}\left(t\right), F\right)\in [\mathrm{0,1}]$ given a probability distribution $F\in P$ on the future space ${R}^{d}$, or to the empirical distribution ${F}_{n}\in P$ obtained from a sample of human capital index values $X\left(t\right)=\{{X}_{1}\left(t\right), \dots {X}_{16}\left(t\right)\}$ and innovativeness index $Y\left(t\right)=\{{Y}_{1}\left(t\right), \dots , {Y}_{16}\left(t\right)\}$,

5 $$D\left( {X_{i} \left( t \right), X\left( t \right)} \right) = \frac{1}{{1 + \frac{1}{n}\mathop \sum \nolimits_{i = 1}^{n} w\left( {\left| {X_{i} \left( t \right) - X\left( t \right)} \right|^{2} } \right)}} ,$$

where $n=16,$ the number of provinces, w is an appropriate non-decreasing and continuous on the space $[0,\infty ]$ weight function, and $\left| {} \right|^{2}$ denotes the Euclidean metric.

In the local test, the Wilcoxon statistic is estimated in a so-called moving window with a given number of observations in the period ${t}_{j}$, ${t}_{k}$, where ${t}_{j}=\mathrm{1,2},3\dots ,14$, and ${t}_{k}=\mathrm{2,3},\dots , 15$

6 $$S\left( {t_{j} ,t_{k} } \right) = \min \left( {W_{{F\left[ {t_{j} , t_{k} } \right]}}^{ + } , W_{{\left[ {Ft_{j} , t_{k} } \right]}}^{ - } } \right),$$

where ${W}^{+}=\sum_{{d}_{i}>0}{R}_{i}^{\beta }\left(F\left(X({t}_{j}),X({t}_{k})\right)\right)$, ${W}^{-}=\sum_{{d}_{i}<0}{R}_{i}^{\beta }\left( F\left({X(t}_{j}),X( {t}_{k})\right)\right)$ are statistics derived from the sum of ranks for positive and negative deviations, respectively, from the median induced by local depth.

This time, for each province and in each year t, a symmetric probability distribution is computed for which the formula can express the depth ${P}^{{X}_{i}(t)}=\frac{1}{2}{P}^{X\left(t\right)}+\frac{1}{2}{P}^{2{X}_{i}\left(t\right)-X(t)}$ and the point surround is of the form (Paindaveine & Bever, 2013):

7 $$R_{i}^{\beta } (F\left( {X\left( {t_{j} } \right),X\left( { t_{k} } \right)} \right) = \mathop \cap \limits_{\alpha \in A\left( \beta \right)} D_{\alpha } (F\left( {X\left( {t_{j} } \right), X\left( {t_{k} } \right)} \right),$$

where $$A\left(\beta \right)=\{\alpha \ge 0:P[{D}_{\alpha }\left(F\right)\ge \beta \}$$

Defining local depth is made more accessible when a parameter $\beta \in (\mathrm{0,1}]$, is introduced, by which the degree of overlap between the values of the statistics in ${t}_{j}$ and ${t}_{k}$ is determined. The local depth corresponds to the smallest central region containing no less than β of probability masses

8 $$LD^{\beta } \left( {z,P} \right):z \to D\left( {z, P^{{X_{i} \left( t \right)\beta }} } \right)$$

where ${{P}^{{X}_{i}\left(t\right)}}^{\beta } \left(. \right)=P\left(.\right|{R}_{i}^{\beta }(P))$ is a conditional distribution concerning its surroundings${R}_{i}^{\beta }(P)$. For $\beta =1,$ local depth is reduced to mere global depth.

### Data Set—Effectiveness of Cohesion Policy in Reducing Regional Disparities

The assessment of the impact of the EU Operational Programs Human Capital and Innovative Economy operating in 2007–2013, and the Knowledge, Education, Development (2014–2020) as well as the earlier sectoral programs (e.g., Integrated Regional Operational Program, IROP) from 2004 to 2006, was based on the concept of functional data. In the testing, it was assumed that the level of human capital development and innovation potential was a derivative of the previously selected partial variables. According to the study's methodology, each voivodeship was seen separately pursuing the same human capital development model or innovation. All regions formed a set of functional data. The effectiveness of EU programs in increasing the socio-economic potential of Polish regions, especially in human capital and innovativeness, can be checked using composite indices in over the period 2004–2018. We adopt the Wilcoxon procedure for functional data to check if there were any changes in the research period at the beginning of the study and fourteen years later.

## Results

**Fig. 1:**
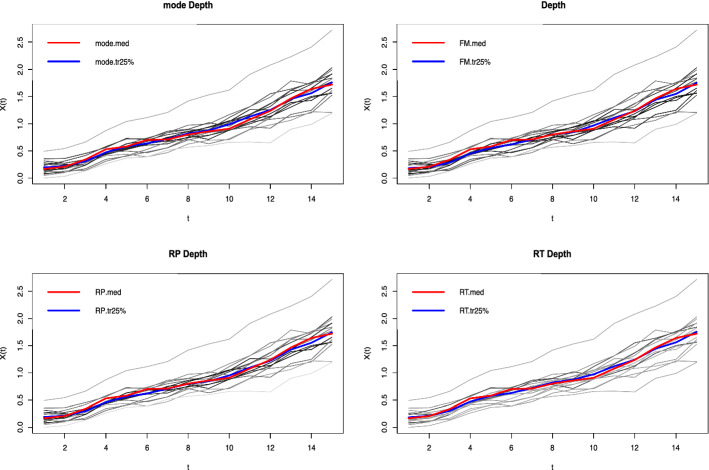
Average path of human capital development of provinces in 2004–2018, with four ways of calculating the median. Source: own elaboration. Calculations were performed using the R language

**Fig. 2:**
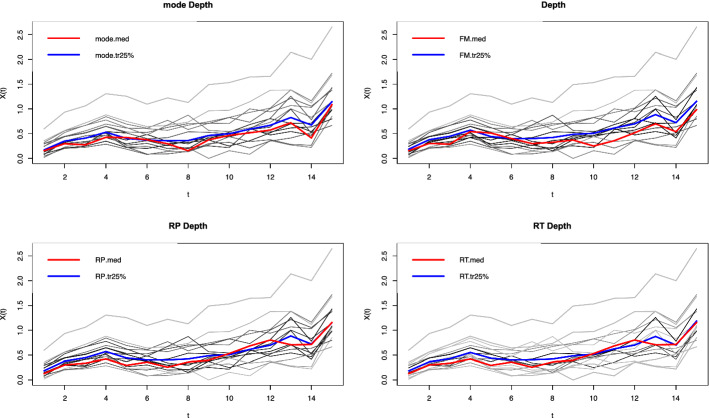
Average path of innovation development of provinces in 2004–2018, with four ways of calculating the median. Source: own elaboration. Calculations were performed using the R language

EU cohesion policy's effectiveness on human capital development and innovation in Polish voivodeships are presented in Tables 1 and 2. The trajectory of Polish voivodeships' human capital and innovation development with the median value is shown in Figs. 1 and 2. The red line shows the average path of human capital development in the voivodeships (the functional median). The blue line, in turn, is the average path of development, with the 25% most outlier observations omitted.

**Table 1:** The results of the Wilcoxon global test. *Source* own study in R language using the depthProc package

	State of human capital (2004–2005) vs. 2017–2018	State of innovativeness (2004–2005) vs. 2017–2018
Statistics	126.5	128.0
*p*-value	0.9699	0.9999

**Table 2:** Local Wilcoxon test results. *Source* own elaboration in R language using depthProc package. The base period covered the years 2004–2005

Testing period	Human capital		Innovativeness	
	Statistics	*p*-value	Statistics	*p*-value
2005–2006	119	0.6380	99	0.8677
2006–2007	116	0.6812	100	0.8587
2007–2008	164	0.0901	71	0.9852
2008–2009	130	0.4774	102	0.8413
2009–2010	85.5	0.9480	133	0.4326
2010–2011	104	0.8222	112	0.7330
2011–2012	90	0.9268	173	0.0467
2012–2013	139	0.3461	145	0.2669
2013–2014	121	0.6115	173	0.0469
2014–2015	127.5	0.5150	200	0.0029
2015–2016	126	0.5375	183	0.0194
2016–2017	93.5	0.9066	172	0.0505
2017–2018	106.5	0.7972	135	0.4046

Comparing the human capital and innovation in the initial period of the study, i.e., 2004–2005, and the results in the final phase, i.e., 2017–2018, the dispersion of provinces did not change. The study in the local test case aimed to check the differences between the initial state of human capital and innovation in 2004–2005, defined as the reference period, and the test periods, which included consecutive years from 2005 to 2018.

The local test results in Table 2 indicate that the dispersion of voivodeships by human capital decreased statistically in a significant manner only in 2007–2008. We assume it has resulted from Poland's accession to the European Union and the National Development Plan for 2004–2006, especially its implementation programs, i.e., the Integrated Regional Operational Program 2004–2006 and the Sectoral Operational Program Human Resources Development. Since 2007 also OP Human Capital started to operate.

In the case of innovation status, the dispersion of voivodeships decreased between 2011 and 2012, associated with the OP Innovative Economy, and 2013 and 2017. Short-term reduction of disproportions in the latter period is associated with the entry into force of programs under the EU's new financial perspective (2014–2020), i.e., OP Knowledge Education Development, OP Intelligent Development, OP Digital Poland, Horizon 2020, Cosme, Erasmus + . The funds for regional programs were increased (from 25 to 40% of total funds).

## Discussion

Adjustment processes showed a short-term character, which means that, on average, over the entire period, the level of inequality in human capital and innovativeness did not decrease. The problem can be the heavily industrialized and still notably agrarian structure of the Polish economy, which is based on labor-intensive sectors and consists mainly of micro, small, and medium-sized companies. Moreover, company development strategies are dominated by opportunism and acting on short-term goals. The lack of success in reducing disparities in human capital and innovation is also an aftermath of Poland's polarization and diffusion of development policy. This model has changed since 2015 after the election of a new government, which has set as a development priority the equalization of opportunities, help for smaller towns, and social, economic, and territorial cohesion. However, it is too early to assess what effect this paradigm shift has had on the development disparities of Polish voivodeships.

The results of our research confirmed that the literature on the subject is not conclusive (Biedka et al., 2022; Darvas et al., 2019; Fiaschi et al., 2018). The evaluation of cohesion policy can vary depending on what aspect we study, how we frame the problem, and what methodology we use. We agree that the cohesion policy has increased the growth rate of the country as a whole, but it has not reduced regional disparities in two important factors: human capital and innovation. This is important information for politicians and public authorities, as it opens up a discussion on how to then eliminate regional disparities to make development more sustainable. During further research, we will attempt to identify which factor in human capital most influences regional disparities.

The reason for the current developmental disproportions between Polish regions is the historical and geographical accumulation of human capital (Czyż & Hauke, 2011; Gorzelak, 2006; Gurgul & Łach, 2019; Jagódka, 2021; Jagódka & Snarska, 2021; Opiłowska, 2019; Wielki et al., 2018). It turns out that large cities are more successful in applying for public support, which means that weaker regions still lack development impulses for smaller cities and urban areas. We have shown the same as in the literature that human capital is regionally differentiated (Bronzini & Piselli, 2009; Rodríguez-Pose & Vilalta-Bufí, 2005) and is the main factor explaining regional disparities (Erdem, 2016).

Based on the Wilcoxon test and local and global data depth, the regional convergence processes in human capital and innovation showed only short-term character. In the medium term, they did not decrease. The implemented European cohesion policy has proven to be ineffective. One of the reasons was the low budget devoted to regional CP (about 1/3 of the total budget, representing about 1% of the Union's GDP). Other reasons include the Polish business sector's weakness and economy, where heavy industry and agriculture still have an outstanding share.

Cohesion policy has undoubtedly contributed positively to economic growth. However, unfortunately, it has not reduced regional disparities in human capital and innovation. Compensatory processes concerning the average growth path were only temporary. This means that the EU funds stimulated only initially, but not permanently. This is not an indictment of the ineffectiveness of CP, quite the contrary. For the adjustment speed of the underdeveloped regions to be faster, the support measures under CP should be much larger.

Currently, the EU budget represents only about 1% of its GDP, and cohesion policy resources for 2014–2020 represent about 1/3 of the total resources. It is challenging to obtain a consensus on this issue because countries like France and Germany are unwilling to participate more in support programs. It is difficult for the governments of these countries to explain to their citizens that CP helps all regions, including net contributors, mainly by increasing their markets and access to skilled, cheap labor. The skeptical attitude of net contributor states towards CP has intensified with the exit of the UK from the EU.

Regional disparities can be removed through development programs with an adequate critical mass of CP. This means that resources for CP should not be reduced (as advocated by opponents of the convergence policy) but significantly increased so that the adjustment processes take material form.

A certain limit to our survey is the range of features, which in this case is broad, due to the availability of data from the Polish statistical office. It is uncertain whether the same range of data will be available for other countries. In addition, Polish voivodeships are not homogeneous, and often within them, there is additional differentiation. A glaring example is the Mazowieckie Voivodeship. It is a model province, while only the capital Warsaw and its neighboring towns achieve strong results, while the rest of the region ranks last in the country in various surveys. However, we use some regional averaging, which is a simplification for studying differences between regions but does not capture all the causes of disparities. After all, the fact that metropolises and cities, in general, stand out from the rest of the areas (small towns and rural areas) is a fact. In our opinion, this is, among other things, a derivative of the regional distribution of human capital.

As far as future challenges, we should focus on good assessment of the high location of sustainable development and human capital in the development strategies of the European Union. Human capital, which tends to have an uneven regional distribution, accounts for most of the inequality. This is all the more relevant in the current times. We have successive waves of industrial revolutions where uncertainty in the labor market is growing, and new labor relations and competencies are emerging. Moreover, the COVID-19 pandemic has changed today's approach to human capital in many areas. It has been noted that health capital is also relevant and essential for national security. The pandemic has accelerated digitization processes. The challenge for public authorities is, therefore, to counteract social exclusion. There is no denying that the importance of cohesion policy has increased during the COVID-19 pandemic. We consider to include that impact in our future research.

Nationalist sentiments emerge in Europe due to the pandemic, compounded by wage and income disparities. Therefore, it is crucial to increase resources under the CP and place human capital and innovation high in the areas of public intervention. Otherwise, policymakers face increasing public dissatisfaction and more left behind places.

## Conclusion

Development programming is a manifestation of an active policy of public authorities in the determination of development directions. Poland's development planning has increased in importance after the country acceded to the European Union. Currently, it is an obligatory criterion for the allocation of support measures under the cohesion policy.

Regional disparities within human capital and innovativeness and the European Union's cohesion policy were the subjects of attention. The evaluation of the effectiveness of the European CP in the Polish regions in the period 2004–2018 in the context of regional convergence of provinces due to the state of human capital and innovation was carried out through functional data analysis. Cohesion policy final was mainly implemented through the program of the period 2004–2006: IROP, 2007–2013: OP Human Capital, OP Innovative Economy, and 2014–2020: OP Knowledge Education Development, OP Intelligent Development, OP Digital Poland. As a result of the Wilcoxon test and the use of the method of the depth of local and global data, it was found that the regional convergence processes in human capital and innovation showed only short-term character, and in the medium term did not decrease.

## Supplementary Information

Below is the link to the electronic supplementary material.

Supplementary file1 (DOCX 47 kb)

## Data Availability

The datasets generated during and/or analysed during the current study are available from the corresponding author on reasonable request.
