# Supplementary material for: Should We Continue EU Cohesion Policy? The Dilemma of Uneven Development of Polish Regions
Source: Soc Indic Res. 2022 Dec 16;165(3):901–17. doi: 10.1007/s11205-022-03048-8 (PMC9756696; doi:10.1007/s11205-022-03048-8)
Supplement: Supplementary file 1 — Supplementary file1 (DOCX 47 kb) [file 11205_2022_3048_MOESM1_ESM.docx]

Appendix A

The symbol X_ijz, i stands for human life stages (childhood, school, adulthood, old age). Additionally, j is the number of the sub-area of research variables, z specifies the two-digit number of the variable in the associated group.

The symbol Y_ij, i stands for a certain innovativeness area (innovativeness of entrepreneurs, export/sales of innovative products, foreign direct investments, potential of science, inventiveness, and economic growth). Additionally, j is the two-digit number specifying the variable in the associated group.

**Table A1.** Variables for regional human capital.

| **Area** | **Sub** | **S/D** | **Variable** | **Name** | **Unit** | **Source** |
| --- | --- | --- | --- | --- | --- | --- |
| Childhood |  | D | X_1001_ | Infant mortality rate | % | Eurostat |
|  |  | S | X_1002_ | Life expectancy at birth | year | Eurostat |
|  |  | S | X_1003_ | Number of midwives per 10,000 inhabitants | person | LDB |
|  |  | S | X_1004_ | Fertility rate | % | LDB |
|  |  | S | X_1005_ | Children in nursery | % | LDB |
|  |  | S | X_1006_ | Children in pre-school education establishments per 1 thousand children aged 3–5 | person | LDB |
|  |  | S | X_1007_ | Average monthly disposable income per person | PLN | LDB |
| Schooling time/education |  | S | X_2001_ | Gross enrolment ratio—primary schools | % | LDB |
|  |  | S | X_2002_ | Gross enrolment ratio—lower secondary schools | % | LDB |
|  |  | S | X_2003_ | Gross enrolment ratio—general secondary schools | % | LDB |
|  |  | S | X_2004_ | Gross enrolment ratio—vocational schools | % | LDB |
|  |  | S | X_2005_ | Gross enrolment ratio—technical secondary schools | % | LDB |
|  |  | S | X_2006_ | Gross enrollment ratio in higher education | % | LDB |
|  |  | S | X_2007_ | Mean years of schooling | year | UNDP |
|  |  | D | X_2008_ | Early leavers from education and training | % | Eurostat |
|  |  | S | X_2009_ | Students per 10,000 inhabitants | person | LDB |
|  |  | S | X_2010_ | Percentage of students majoring in technical and natural sciences | % | LDB |
|  |  | S | X_2011_ | Graduates per 10,000 population | person | LDB |
|  |  | S | X_2012_ | Postgraduate students per 10 thousand inhabitants | person | LDB |
|  |  | S | X_2013_ | Doctoral students per 10 thousand inhabitants | person | LDB |
|  |  | S | X_2014_ | Number of academic teachers per 10 thousand inhabitants | person | LDB |
|  |  | S | X_2015_ | Number of academic teachers with professorial title per 10 thousand inhabitants | person | LDB |
|  |  | D | X_2016_ | Number of students per academic teacher | person | LDB |
|  |  | D | X_2017_ | Number of pupils per section in primary schools | person | LDB |
|  |  | D | X_2018_ | Number of pupils per section in lower secondary schools | person | LDB |
|  |  | D | X_2019_ | Number of pupils per section in general secondary schools | person | LDB |
|  |  | D | X_2020_ | Number of pupils per teacher in primary school | person | LDB |
|  |  | D | X_2021_ | Number of pupils per teacher in lower secondary schools | person | LDB |
|  |  | D | X_2022_ | Number of pupils per teacher in general secondary schools | person | LDB |
|  |  | D | X_2023_ | Number of pupils per teacher in technical secondary schools | person | LDB |
|  |  | D | X_2024_ | Number of pupils per teacher in vocational schools | person | LDB |
|  |  | S | X_2025_ | Percentage of students additionally learning a foreign language in primary schools | % | LDB |
|  |  | S | X_2026_ | Percentage of students additionally learning a foreign language in lower secondary schools  Students following additional foreign language instruction | % | LDB |
|  |  | S | X_2027_ | Gymnasium exams—the average performance of students (in the mathematics and natural sciences part) | % | LDB |
|  |  | S | X_2028_ | Gymnasium exams—the average performance of students (in the humanities part) | % | LDB |
|  |  | S | X_2029_ | Passing the exam maturity examination in relation to the national average | % | LDB |
|  |  | S | X_2030_ | Average monthly per capita household expenditure on education | PLN | LDB |
|  |  | S | X_2031_ | Expenditure on education as a proportion of total household expenditure | % | LDB |
|  |  | S | X_2032_ | Expenditures of territorial self-government units on education per student | PLN | LDB |
|  |  | S | X_2033_ | Expenditure on higher education per student | PLN | SP |
| Adulthood | Education | D | X_3101_ | Percentage of people participating in lifelong learning aged 25–64 | % | Eurostat |
|  |  | S | X_3102_ | Percentage of people aged 25–64 with primary education | % | Eurostat |
|  |  | S | X_3103_ | Percentage of people aged 25–64 with secondary education | % | Eurostat |
|  |  | S | X_3104_ | Percentage of people aged 25–64 with tertiary education | % | Eurostat |
|  | Demographic  potential | S | X_3201_ | Population density | person/km^2^ | LDB |
|  |  | S | X_3202_ | The net change in population, adjusted for migration | person | LDB |
|  |  | S | X_3203_ | The working-age population to the total population | % | LDB |
|  |  | D | X_3204_ | Demographic dependency ratio for the population in post-working age | % | LDB |
|  |  | D | X_3205_ | The median age of the population | year | Eurostat |
|  | Job | D | X_3301_ | Percentage of registered unemployed with tertiary education | % | LDB |
|  |  | D | X_3302_ | Percentage of unemployed with post-secondary and secondary technical education | % | LDB |
|  |  | D | X_3303_ | Percentage of unemployed registered with general secondary education | % | LDB |
|  |  | D | X_3304_ | Percentage of unemployed with vocational education |  | LDB |
|  |  | D | X_3305_ | Percentage of unemployed with primary education |  | LDB |
|  |  | D | X_3306_ | Share of the long-term unemployed in the total population of the unemployed | % | Eurostat |
|  |  | D | X_3307_ | Youth unemployment rate (population aged 15–24) | % | Eurostat |
|  |  | D | X_3308_ | Economically inactive per 10 thousand economically active | % | GUS |
|  |  | D | X_3309_ | Percentage of job vacancies | % | GUS |
|  |  | D | X_3310_ | Percentage of people not in work or education aged 15–24 | % | Eurostat |
|  |  | D | X_3311_ | Duration of job search | month | LDB |
|  |  | S | X_3312_ | Young people neither in employment nor in education and training | % | Eurostat |
|  |  | S | X_3313_ | The employment rate for people with primary education | % | Eurostat |
|  |  | S | X_3314_ | The employment rate of people with secondary education | % | Eurostat |
|  |  | S | X_3315_ | The employment rate for people with tertiary education | % | Eurostat |
|  |  | S | X_3316_ | Employment rates by age 15–24 | % | Eurostat |
|  |  | S | X_1317_ | Employment rates by age 25–34 | % | Eurostat |
|  |  | S | X_1318_ | Employment rates by age 35–44 | % | Eurostat |
|  |  | S | X_1319_ | Employment rates by age 45–54 | % | Eurostat |
|  |  | S | X_1320_ | Employment rates by age 55–64 | % | Eurostat |
|  |  | S | X_3321_ | Economic activity rates by primary education | % | Eurostat |
|  |  | S | X_3322_ | Economic activity rates by secondary education | % | Eurostat |
|  |  | S | X_3323_ | Economic activity rates by tertiary education | % | Eurostat |
|  |  | S | X_3324_ | The employment rate of disabled people aged 16–64 | % | LDB |
|  |  | S | X_3325_ | Average number of usual weekly hours of work in the main job by age 15–24 | hour | Eurostat |
|  |  | S | X_3326_ | Average number of usual weekly hours of work in the main job by age 25–64 | hour | Eurostat |
|  |  | S | X_3327_ | Average number of usual weekly hours of work in the main job by age 65–74 | hour | Eurostat |
|  |  | S | X_3328_ | Gross value added per employee | PLN | LDB |
|  |  | S | X_3329_ | Average gross salary of persons with higher education (M.A. and higher) | % | SP |
|  |  | S | X_3330_ | Average gross salary of persons with higher bachelor’s and engineer’s degrees in comparison to the national average | % | SP |
|  |  | S | X_3331_ | Average gross salary of persons with post-secondary education in comparison to the national average | % | SP |
|  |  | S | X_3332_ | Average gross salary of persons with technical education in comparison to the national average | % | SP |
|  |  | S | X_3333_ | Average gross salary of persons with general secondary education in comparison to the national average | % | SP |
|  |  | S | X_3334_ | Average gross salary of persons with general vocational education in comparison to the national average | % | SP |
|  |  | S | X_3335_ | Average gross salary of persons with lower secondary education in comparison to the national average | % | SP |
|  |  | S | X_3336_ | Average gross salary of persons with primary and incomplete primary education in comparison to the national average | % | SP |
|  | R&D and KBE | S | X_3401_ | Expenditures on innovation activity in enterprises per economically active person | PLN | LDB |
|  |  | S | X_3402_ | R&D expenditure per capita | PLN | LDB |
|  |  | S | X_3403_ | Employment in technology and knowledge-intensive sectors to the total number of employees | % | Eurostat |
|  |  | S | X_3404_ | Human resources in science and technology (HRST) as % of the active population | % | Eurostat |
|  |  | S | X_3405_ | Employment in high-tech sectors as % of the active population | % | Eurostat |
|  | Entrepreneurship | S | X_3501_ | Entities entered in the REGON register per 10 thousand population | object | LDB |
|  |  | S | X_3502_ | Natural persons conducting economic activity per 100 persons of working age | person | LDB |
|  |  | S | X_3503_ | Number of business environment institutions per 100 thousand inhabitants |  | IOB |
|  |  | S | X_3504_ | Funds from E.U. to finance programs and projects E.U. per capita | PLN | LDB |
|  |  | S | X_3505_ | Households furnished with a personal computer and a broadband connection to the Internet | % | LDB |
|  | Social capital | S | X_3601_ | Voter turnout in parliamentary elections | % | LDB |
|  |  | S | X_3602_ | Foundations, associations, and social organizations per 10, 000 population | object | LDB |
|  | Leisure | S | X_3701_ | Library loans per borrower in volumes | book | LDB |
|  |  | D | X_3702_ | Number of population per one seat in fixed cinemas | person | LDB |
|  |  | S | X_3703_ | Museum/branch visitors per 10, 000 population | person | LDB |
|  |  | S | X_3704_ | Sports clubs including religious and UKS clubs per 10, 000 inhabitants | object | LDB |
|  | Social exclusion | D | X_3801_ | Social assistance benefits—beneficiaries per 10, 000 population | PLN/person | LDB |
|  |  | D | X_3802_ | Poverty rates—in % of persons in households with expenditures below the extreme poverty threshold | % | LDB |
|  | Health | S | X_3901_ | Nurses and midwives per 10 thous. population | person | LDB |
|  |  | S | X_3902_ | Doctors per 10 thous. population | person | LDB |
|  |  | S | X_3903_ | Beds in general hospitals per 10 thous. population | object | LDB |
|  |  | D | X_3904_ | Deaths of people due to cardiovascular disease per 100 thous. population | person | LDB |
|  |  | D | X_3905_ | Deaths due to cancer per 100 thous. population | person | LDB |
|  |  | D | X_3906_ | Deaths of people due to mental and behavioral disorders per 10 thous. population | person | LDB |
|  |  | D | X_3907_ | Suicide per 10 thous. population | person | LDB |
|  |  | D | X_3908_ | Occupational diseases per 10 thous. economically active persons | person | LDB |
|  |  | S | X_3909_ | Average monthly gross pension due to an inability to work | PLN | LDB |
|  |  | S | X_3910_ | Private households—average monthly expenditures on health per capita | PLN | LDB |
|  |  | S | X_3911_ | Local government expenditures on health care per capita |  | LDB |
| Postproductive  age |  | S | X4001 | Average monthly gross retirement from a non-agricultural social security system | PLN | LDB |
|  |  | S | X4002 | Average monthly gross retirement for farmers | PLN | LDB |
|  |  | S | X4003 | Economic activity rates after 65 years old | % | Eurostat |
|  |  | S | X4004 | Residents of social welfare home per 10 thous. inhabitants | person | LDB |

Source: own elaboration based on public sources.

**Table A2.** Variables describing regional innovativeness.

| **Area** | **Variable** | **Name** | **Unit** | **Source** |
| --- | --- | --- | --- | --- |
| Innovativeness of enterprises | Y_101_ | The average share of innovative enterprises in the total number of enterprises | % | LDB |
|  | Y_102_ | Innovative service sectors enterprises by new or improved products | % | LDB |
|  | Y_103_ | Innovative service sectors enterprises by new or improved products to the market | % | LDB |
|  | Y_104_ | Innovative service sectors enterprises by new or improved business processes | % | LDB |
|  | Y_105_ | Innovative industrial enterprises by new or improved products | % | LDB |
|  | Y_106_ | Innovative industrial enterprises by new or improved products to the market | % | LDB |
|  | Y_107_ | Industrial enterprises by new or improved business processes | % | LDB |
|  | Y_108_ | Innovative industrial enterprises that cooperated in terms of innovation activity in % of total enterprises | % | LDB |
|  | Y_109_ | Innovative enterprises in the service sector that cooperated in terms of innovation activity in % of total enterprises | % | LDB |
|  | Y_110_ | Industrial enterprises that participated in innovation activities cluster or other formal types of cooperation in % of the innovation active enterprises | % | LDB |
| Sales/export of innovative products | Y_201_ | Share of net revenues from sales of exported innovative products to the market in total net revenues from sales (industrial enterprises) | % | LDB |
|  | Y_202_ | Share of net revenues from sales of exported innovative products in total net revenues from sales (industrial enterprises) | % | LDB |
|  | Y_203_ | Share of net revenues from sales of innovative to the market products in total net revenues from sales (industrial enterprises) | % | LDB |
|  | Y_204_ | Share of sold production of new or significantly improved industry goods in industrial enterprises in the total amount of sold goods | % | LDB |
|  | Y_205_ | Share of net income from the sale of products in entities classified to high and medium- | % | LDB |
| FDI foreign direct investment | Y_301_ | New entities of the national economy recorded per 10, 000 of population at working age | object | LDB |
|  | Y_302_ | Investment outlays in the companies with foreign capital to total investment outlays | % | LDB |
|  | Y_303_ | Foreign capital per inhabitant of working age | PLN | LDB |
|  | Y_304_ | Number of units with foreign capital per 100 thousand inhabitants | unit | LDB |
|  | Y_305_ | Number of employees in companies with foreign capital participation per 1 thousand total employees | unit | LDB |
| inventiveness | Y_401_ | Patents granted by the Patent Office of the Republic of Poland (PORP) per 100 thous. population | unit | PORP |
|  | Y_402_ | Rights of protection granted by the Patent Office of the Republic of Poland per 100 thous. population | unit | PORP |
|  | Y_403_ | Patent applications filed in the Patent Office of the Republic of Poland per 100 thous. population | unit | PORP |
|  | Y_404_ | Rights of protection filed in the Patent Office of the Republic of Poland per 100 thous. population | unit | PORP |
|  | Y_405_ | Patents-applications filed with EPO (Patstat) per 100 thous. inhabitants | unit | Patstat |
| Potential of science | Y_501_ | Scientific publications per 1 million inhabitants with at least one foreign author in English | unit | RIS |
|  | Y_502_ | Number of scientific publications with the highest citations (10% of the most citations worldwide) to the total number of publications in the region as a relation to the E.U. average | unit | RIS |
| Economic Growth | Y_601_ | Gross domestic product per capita | PLN | LDB |

Source: own elaboration based on public sources.
